# Functional physiological, psychological, and biochemical reactivity to socially evaluated cold pressor test in hereditary angioedema patients (FRoSEn)

**DOI:** 10.3389/fimmu.2025.1736589

**Published:** 2026-01-07

**Authors:** Beatrice De Maria, Luca Ranucci, Clara Gino, Azzurra Cesoni Marcelli, Lorenza Chiara Zingale, Aida Zulueta, Laura Adelaide Dalla Vecchia, Alessandra Gorini, Francesca Perego

**Affiliations:** 1 Istituti Clinici Scientifici Maugeri IRCCS, Bioengineering Laboratory, Milan, Italy; 2 Istituti Clinici Scientifici Maugeri IRCCS, PsyCaRe Lab – Laboratorio di Psicologia per un Approccio Integrato alle Patologie Cardiopolmonari e le Malattie Rare in Ambito Cardiovascolare, Milan, Italy; 3 Istituti Clinici Scientifici Maugeri IRCCS, Department of Internal Medicine and Rehabilitation, Milan, Italy; 4 Istituti Clinici Scientifici Maugeri IRCCS, Laboratorio di Ricerca sui Biomarcatori Neurologici (LaBioN), Milan, Italy; 5 Istituti Clinici Scientifici Maugeri IRCCS, Department of Cardiology, Milan, Italy; 6 Università degli Studi di Milano, Dipartimento di Scienze Cliniche e di Comunità, Dipartimento di Eccellenza 2023-2027, Milan, Italy

**Keywords:** blood pressure, heart rate, hereditary angioedema, inflammation, rare disease, secondary prevention, Socially Evaluated Cold Pressor Test, stress

## Abstract

**Introduction:**

Stressful physical or psychological events can trigger acute swelling attacks in patients with Hereditary Angioedema due to C1 Inhibitor deficiency (HAE-C1INH), although the stress–disease relationship remains unclear. The Socially Evaluated Cold Pressor Test (SECPT) reliably induces acute stress under controlled conditions. This study aimed to compare perceived stress, inflammatory markers, and cardiovascular responses to SECPT between HAE-C1INH patients and healthy controls (HC).

**Methods:**

Twenty HAE-C1INH patients (9 males, 44 ± 14 years) and age and sex matched HC underwent a 3-minute SECPT. Participants completed questionnaires assessing anxiety and depression (HADS), pain catastrophizing (PCS), and subjective stress (0–100 scale) before and after SECPT. Heart rate (HR) and arterial pressure (AP) were recorded. Blood samples for inflammatory cytokines (IL-6, IL-1ß, TNF-α) were collected at baseline, and 10 and 40 minutes after SECPT.

**Results:**

Compared to HC, patients showed higher baseline HADS-A (7.3 ± 4.5 vs 4.7 ± 2.7), overall PCS (19.7 ± 12.6 vs 12.9 ± 8.7), and perceived stress during SECPT (60.6 ± 34.3 vs 34.6 ± 23.8). IL-6 levels were higher at baseline and 10 minutes post-test (2.63 ± 1.21 vs 1.84 ± 0.87; 2.78 ± 1.20 vs 1.91 ± 0.79 pg/ml), as were TNF-α levels across all phases (4.19 ± 1.38 vs 3.26 ± 1.55; 4.09 ± 1.39 vs 3.40 ± 1.48; 4.09 ± 1.28 vs 3.20 ± 1.57) while IL-1 ß remained unchanged. HR and AP variations were similar between groups.

**Discussion:**

HAE-C1INH patients exhibited heightened perceived stress response to SECPT, and elevated baseline inflammation, despite comparable cardiovascular reactivity. These findings highlight a complex psychophysiological–inflammatory interplay in acute stress responses, suggesting the need to integrate psychological and biological frameworks in understanding HAE-C1INH triggers.

**Clinical trials code:**

NCT06414252.

## 1 Introduction

Hereditary angioedema due to C1 inhibitor deficiency (HAE-C1INH Type 1 and Type 2) is a rare (prevalence 1:65000) and potentially life-threatening disease due to the dysfunction of C1-inhibitor (C1-INH) protein, a key regulator of the complement, contact, and fibrinolytic systems. HAE-C1INH patients experience recurrent episodes of localized edema affecting the extremities, bowel mucosa, face, and upper airways (1–4). Stressful physical and psychological events are frequently reported by patients to trigger acute HAE attacks (5–7). However, this evidence has never been investigated under controlled conditions and is mainly derived from the patients’ narratives (8).

While the molecular mechanisms underlying HAE-C1INH are well described, the interplay between stress response, cardiovascular control, inflammation and symptoms manifestation remains poorly understood (8–10). Physical and psychological stress acts on the hypothalamic–pituitary–adrenal (APA) axis and autonomic nervous system (ANS), leading to the release of stress and inflammatory biomarkers and activation of the sympathetic branch of the ANS, potentially influencing the vascular permeability and inflammatory response of HAE-C1INH patients (11, 12). For these reasons, the evaluation of the interaction between the ANS and inflammatory system should be integrated with the study of the psychological response to stress in HAE-C1INH patients, as stress-induced changes could exacerbate or modulate disease symptoms (13–15).

The Socially Evaluated Cold Pressor Test (SECPT) is a well-established method for inducing acute physical and psychological stress in laboratory settings (16). According to cognitive appraisal models (17), stress responses depend on the perceived balance between situational demands and coping resources. Thanks to its social-evaluative component, SECPT triggers heightened affective and physiological activation through perceived threat and loss of control (18) and could offer a deeper insight into the mechanisms by which stress influences physiological, biochemical and mental response in HAE-C1INH patients (18).

In addition to physiological and biochemical indices, the assessment of psychological variables provides essential insight into how patients perceive and regulate stress. Individual differences in anxiety, perceived stress, body awareness, and pain-related cognitions can significantly influence the magnitude and direction of physiological stress responses (19, 20). For instance, higher levels of anxiety or pain catastrophizing are associated with increased sympathetic activation and proinflammatory signaling, whereas greater interoceptive and body awareness may promote adaptive stress regulation (21, 22). Evaluating these psychological dimensions in HAE-C1INH patients allows a more comprehensive understanding of how cognitive–emotional factors interact with autonomic and inflammatory mechanisms in shaping stress reactivity. This integrative approach may ultimately clarify whether psychological vulnerability contributes to the heightened stress sensitivity and the potential triggering of attacks observed in this population.

The aim of the present study was to provide a multidimensional characterization of the stress response to SECPT in patients with HAE-C1INH, analyzing arterial blood pressure, heart rate, inflammatory cytokine levels, and perceived stress before, during and after the exposure to SECPT, and to compare these responses with those of a group of matched healthy controls (HC).

## 2 Methods

### 2.1 Population

HAE-C1INH patients were consecutively enrolled during ambulatory visits from June 2023 to April 2024 at Istituti Clinici Scientifici Maugeri, IRCCS, Milan, Italy. The inclusion criteria were: i) confirmed diagnosis of HAE-C1INH according to guidelines (5); ii) age between 18 and 65 years; iii) ability to provide a written informed consent. The exclusion criteria were: i) any type of chronic diseases requiring chronic treatment (i.e. hypertension, previous myocardial infarction, diabetes, chronic heart failure, autoimmune disease, neurodegenerative diseases); ii) active acute diseases; iii) Sars-Cov2 infection in the previous 3 months (23); iv) acute HAE attacks in the 7 days preceding the experimental procedure; v) acute HAE attacks in the 72 hours after the study participation (a posteriori exclusion). Age- and gender- matched HC were recruited between healthcare professionals and their relatives. Non-HAE-related inclusion and exclusion criteria were applied to select the HC group.

The sample size was calculated over the systolic arterial blood pressure variations from REST phase to SECPT (see experimental protocol below) based on data derived from Schwabe et al. (16). Assuming 80% power, a two-sided α of 0.05, and a 1:1 allocation between HAE-C1INH patients and HC, the sample size calculation indicated a minimum of 17 participants per group.

### 2.2 Experimental protocol

The experimental protocol was approved by the Ethics Committee of Istituiti Clinici Scientifici Maugeri (approval number 2774 CE; date of approval 31 May 2023) and was registered on ClinicalTrials.gov (NCT06414252). The study was compliant with the Declaration of Helsinki principles and each subject signed a written informed consent.

**Figure 1:**
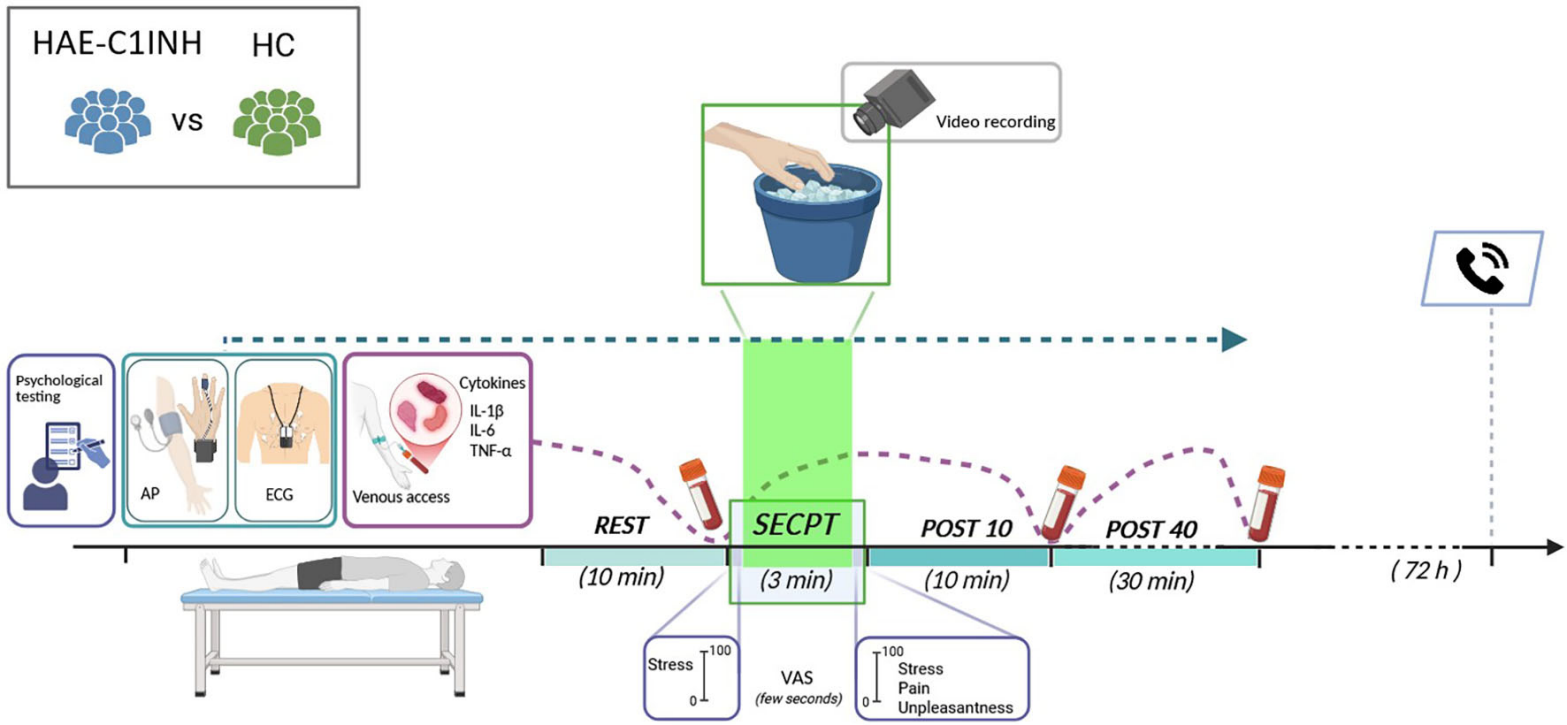
Schematic representation of the experimental protocol. Figure was created in Biorender.com.

An overview of the experimental protocol is provided in **Figure 1**.

All sessions were conducted between 9:00 and 12:00 a.m. in a quiet room with a comfortable temperature to minimize circadian variability of all the registered variables. All participants were instructed to refrain from caffeine or exercise before testing. All the participants underwent a medical visit to verify the inclusion and exclusion criteria and to collect demographic and clinical data.

#### 2.2.1 Psychological assessment

Before starting the experimental procedure, a set of psychological questionnaires was administered to capture multiple dimensions of stress perception, emotional state, and pain-related cognition, all of which may contribute to stress reactivity and symptom modulation in HAE-C1INH. The Hospital Anxiety and Depression Scale (HADS) (24) was included to evaluate the presence of anxiety and depressive symptoms, which are known to influence both physiological stress responses and inflammatory activity. The Perceived Stress Scale (PSS) (25) assessed the individual’s appraisal of stress in daily life, providing a measure of general stress vulnerability that could modulate acute stress responses during the SECPT. The Pain Catastrophizing Scale (PCS) (26) was used to quantify maladaptive cognitive responses to pain, such as rumination, magnification, and helplessness, that may amplify emotional distress and physiological arousal through sustained activation of the autonomic and inflammatory systems.

Together, these instruments provided a multidimensional psychological profile allowing the integration of emotional and cognitive components of stress reactivity. This approach enabled the investigation whether altered stress perception or maladaptive body-related cognitions could interact with physiological and inflammatory mechanisms in HAE-C1INH patients, potentially contributing to their heightened stress sensitivity and susceptibility to attack triggers.

#### 2.2.2 Experimental procedure

Participants were positioned in supine position and equipped with sensors to record the electrocardiogram (ECG, LAB3 device, Marazza, Monza, Italy) and non-invasive arterial blood pressure (AP) via photoplethysmography device (Finometer, Finapres Medical System) positioned on the middle finger of the right hand. ECG was sampled at 1000 Hz, while AP at 250 Hz. A venous catheter was positioned to allow the blood withdrawal during the test to dose the inflammatory biomarkers.

The experimental procedure lasted about 1 hour per subject and was structured in 4 different phases (**Figure 1**).

*REST phase*: during the first 10 minutes participants lied supine. Continuous ECG and non-invasive AP recording started. At the end of this period a blood sample was collected and participants were asked to rate their perceived level of stress based on a Visual Analogue Scale (VAS) (27) score ranging from 0 (“not stressed at all”) to 100 (“very much stressed”).

*SECPT phase*: during this phase, participants were informed that the entire procedure would be video-recorded for the subsequent analysis of facial expressions. They were asked to immerse their left hand up to the wrist in a container filled with cold water maintained at 4°C for 3 minutes, or until the discomfort became intolerable. To enhance the social-evaluative component, participants were instructed to maintain direct eye contact with a camera placed in front of them throughout the immersion, while being observed in silence by the experimenters. This manipulation is known to amplify stress reactivity by inducing a sense of evaluation and loss of control, which activates both the sympathetic–adrenal–medullary (SAM) system and the hypothalamic–pituitary–adrenal (HPA) axis.

At the end of the SECPT phase, participants were asked to rate on a VAS ranging from 0 (‘‘not at all’’) to 100 (‘‘very much’’) how stressful, unpleasant and painful the SECPT experience has been.

*POST10 phase*: participants remained in a supine position. The cardiovascular signals continued to be recorded, and a blood sample was collected after 10 minutes.

*POST40 phase*: participants continued to lie supine. The cardiovascular signals continued to be recorded, and a blood sample was collected after additional 30 minutes (40 minutes after the end of the SECPT phase).

Peripheral blood (6 mL) was collected via the venous catheter from all participants and drawn into tubes containing EDTA as an anticoagulant. After collection, samples were centrifuged at 2,000 × g for 15 minutes. The resulting plasma was aliquoted into 500 µL in polypropylene tubes and stored at −80°C for subsequent molecular analyses.

After 72 hours patients were contacted by phone to verify if any HAE attack occurred (**Figure 1**).

#### 2.2.3 Signal processing

From the acquired ECG, the beat-to-beat RR interval (RR) series was derived. RR was defined as the temporal distance between two consecutive R peaks detected on the ECG. Heart rate (HR) was calculated as the reciprocal of RR in seconds multiplied by 60. From the acquired AP signal, the systolic AP (SAP) time series was derived. SAP was defined as the maximum of the AP signal inside each RR. Diastolic AP (DAP) beat-to-beat time series was derived as well. DAP was defined as the minimum of the AP signal inside each RR.

For each experimental phase the mean HR, the mean SAP and the mean DAP were calculated. As to REST, SECPT and POST 10 phases, the whole period was considered, while for POST40 only the last 10 minutes of this phase were taken for analysis. HR was expressed in beats per minute (bpm), while SAP and DAP in mmHg.

#### 2.2.4 Cytokines quantification

Plasma concentrations of selected cytokines were quantified using high-sensitivity enzyme-linked immunosorbent assay (ELISA) kits: Interleukin-1 beta (IL-1β; E-HSEL-H0001, ElabScience, USA), Interleukin-6 (IL-6; 950.035.096, Diaclone, France), and Tumor Necrosis Factor-alpha (TNF-α; EIA-4641, DRG, Germany). Assays were performed in 96-well plates according to the manufacturers’ protocols. Briefly, each plate included blanks, standards, controls, and samples, all run in duplicate and incubated according to the manufacturer’s instructions. The calibration curve was plotted using the provided kit standards, and the absolute cytokine levels were calculated accordingly. The mean value of the two measurements (pg/ml) was then reported and used for statistical analysis.

### 2.3 Statistical analysis

Continuous data were presented as mean ± standard deviation, while categorical variables as absolute number and percentage. Continuous demographic and clinical characteristics of HC and HAE-C1INH patients were compared by *Student t test* in case of normal distribution or Mann-Whitney test in case of non-normal distribution. χ2 test was applied for categorical variables. Two-way repeated measures analysis of variance (ANOVA, Holm-Sidak test for multiple comparisons) was performed to verify the differences of AP and HR parameters, psychological variables and levels of inflammatory cytokines between the two groups at the different time points. A p<0.05 was considered significant for all the performed analyses. The statistical analyses were conducted with Sigmaplot software (Systat Software, Inc., Chicago, IL, version 11.0).

## 3 Results

### 3.1 Population

Twenty HAE-C1INH patients (9 males, mean age 44 ± 14 years) and 20 HC individuals (9 males, mean age 45 ± 15 years) were enrolled in the study. The demographic and clinical features are presented in **Table 1**. Nine HAE-C1INH patients experienced attacks in the previous 6 months, 2 had one attack, 4 more than two, and 3 more than three; 18 patients were on long-term prophylaxis (LTP): 15 with lanadelumab, and 3 with subcutaneous C1 esterase inhibitor. Compared to HC, patients had a higher body mass index.

**Table 1:** Demographic and clinical characteristics of the enrolled population.

	HC (n=20)	HAE-C1INH (n=20)
Age, yrs	43.95 ± 14.00	44.75 ± 14.83
Sex, males/females, (n)	9/11	9/11
BMI, kg·m^-2^	22.72 ± 3.41	25.02 ± 3.88*
BMI>25 kg·m^-2^, n (%)	5 (25)	7 (35)
Regular physical activity, n (%)	10 (50)	12 (60)
Physical activity, hrs per week	5.4 ± 3.56	6.96 ± 7.21
Age at HAE-C1INH diagnosis, yrs	–	18.65 ± 12.48
Years from the HAE-C1INH diagnosis, yrs	–	26.35 ± 11.39
Attacks in the last 6 months, n (%)	–	9 (45)
No LTP, n (%)	–	2 (10)
LTP with lanadelumab, n (%)	–	15 (75)
LTP with C1 esterase inhibitor, n (%)	–	3 (15)

HC, healthy controls; HAE-C1INH, patients with hereditary angioedema due to C1 inhibitor deficiency; BMI, body mass index; LTP, long-term prophylaxis. Numerical values are presented as mean ± standard deviation, while categorical values as absolute number (percentage). * indicates p<0.05 vs HC.

In the HC group, two participants did not complete the 3-minute SECPT (a 38-year-old man: 70 s; a 63-year-old woman: 74 s). In the HAE-C1INH patient group, five participants failed to complete the test (a 26-year-old woman: 73 s; a 33-year-old man: 42 s; a 37-year-old woman: 50 s; a 66-year-old woman: 65 s; a 58-year-old woman: 96 s).

### 3.2 Psychological questionnaire

The comparison between results obtained in the psychological questionnaires in patients and HC is presented in **Table 2**. Patients obtained higher scores in the HADS anxiety, in the global score of PCS and its magnification domain. Non-significant differences in the HADS depression and PSS were found between the two groups.

**Table 2:** Psychological characterization of the enrolled population.

	HC (n=20)	HAE-C1INH (n=20)
HADS anxiety	4.7 ± 2.7	7.3 ± 4.5*
HADS depression	1.9 ± 1.5	3.1 ± 3.3
PSS	17.2 ± 6.4	17.1 ± 7.2
PCS	12.9 ± 8.7	19.7 ± 12.6*
Helplessness	4.6 ± 4.0	7.0 ± 6.1
Rumination	6.8 ± 4.3	9.1 ± 4.8
Magnification	1.5 ± 1.2	3.5 ± 2.5*

HC, healthy controls; HAE-C1INH, patients with hereditary angioedema due to C1 inhibitor deficiency; HADS, Hospital Anxiety and Depression Scale; PSS, Perceived Stress Scale; PCS, Pain Catastrophizing Scale. Numerical values are presented as mean ± standard deviation, while categorical values as absolute number (percentage). * indicates p<0.05 vs HC. Note: numbers in the table indicate the score of the questionnaires.

### 3.3 Heart rate and arterial blood pressure response to SECPT

**Figure 2:**
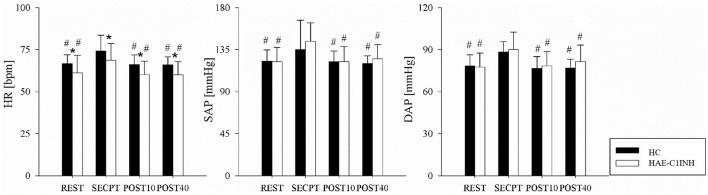
Heart rate and arterial blood pressure in HC and HAE-C1INH patients. The bar graphs show the changes in heart rate (HR), systolic arterial blood pressure (SAP) and diastolic arterial blood pressure (DAP) during the different phases of the experimental protocol: supine resting position (REST), socially-evaluated cold pressure test (SECPT), in the 10 minutes following the SECPT (POST10) and in the 30 minutes following the POST10 (POST40) in HC (black bars) and HAE-C1INH (white bars). Data are presented as mean and standard deviation. * indicates p<0.05 vs HC, # p<0.05 vs SECPT.

The results of the HR, SAP and DAP mean values during REST, SECPT, POST10 and POST40 are reported in **Figure 2**. HR, SAP and DAP increased from REST to SECPT in both patients and HC and decreased in both groups during POST10 and POST40 when compared to SECPT. HR, SAP and DAP values were similar in REST, POST10 and POST40. HR was lower in patients than HC in all the experimental phases.

### 3.4 Inflammatory cytokines quantification

**Figure 3:**
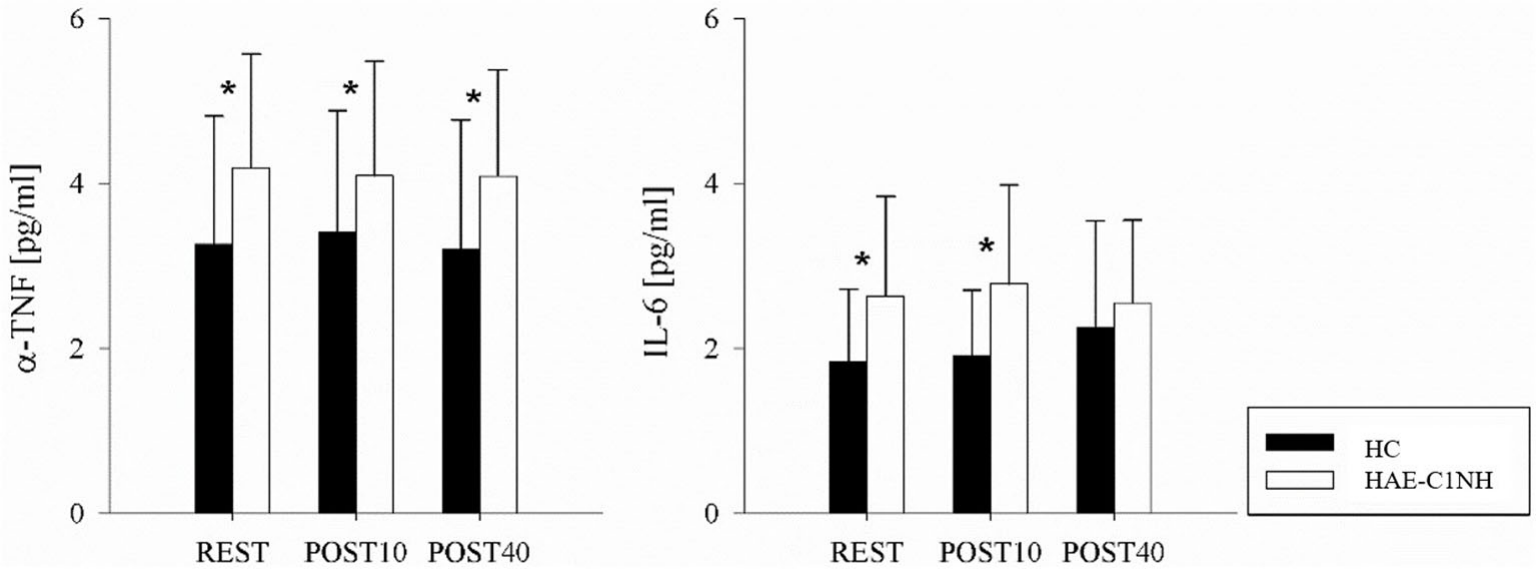
Inflammatory cytokines in HC and HAE-C1INH patients. The bar graphs show the changes in Tumor Necrosis Factor-alpha (TNF-α) and Interleukin-6 (IL-6) at the end of the REST, POST10 and POST40 phases of the experimental protocol in HC (black bars) and HAE-C1INH (white bars). Data are presented as mean and standard deviation. * indicates p<0.05 vs HC

The results of the inflammatory cytokines quantification are presented in **Table 3** and summarized in **Figure 3**. Cytokines quantification was performed in 19 patients and 18 HC. Compared to HC, patients were characterized by higher levels of IL-6 and TNF-α in all the experimental phases (with the exception of IL-6 in POST40). The values of IL-1ß, IL-6 and TNF-α did not vary across the different phases in both groups.

**Table 3:** Inflammatory cytokines quantification.

	HC (n=18)			HAE-C1INH (n=19)		
	*REST*	*POST10*	*POST40*	*REST*	*POST10*	*POST40*
IL-1ß, pg/ml	20.54 ± 26.14	19.79 ± 25.02	20.41 ± 25.61	21.02 ± 28.06	20.61 ± 27.06	20.80 ± 27.57
IL-6, pg/ml	1.84 ± 0.87	1.91 ± 0.79	2.25 ± 1.30	2.63 ± 1.21*	2.78 ± 1.20*	2.55 ± 1.00
TNF-α, pg/ml	3.26 ± 1.55	3.40 ± 1.48	3.20 ± 1.57	4.19 ± 1.38*	4.09 ± 1.39*	4.09 ± 1.28*

HC, healthy controls; HAE-C1INH, patients with hereditary angioedema due to C1 inhibitor deficiency; REST, supine resting position; SECPT, socially-evaluated cold pressure test; POST10, 10 minutes following the SECPT; POST40, 40 minutes following the SECPT; IL-1ß, interleukin-1 beta; IL-6, interleukin-6; TNF-α, Tumor Necrosis Factor-alpha. Values are presented as mean ± standard deviation. * indicates p<0.05 vs HC.

### 3.5 Psychological response to SECPT

**Figure 4:**
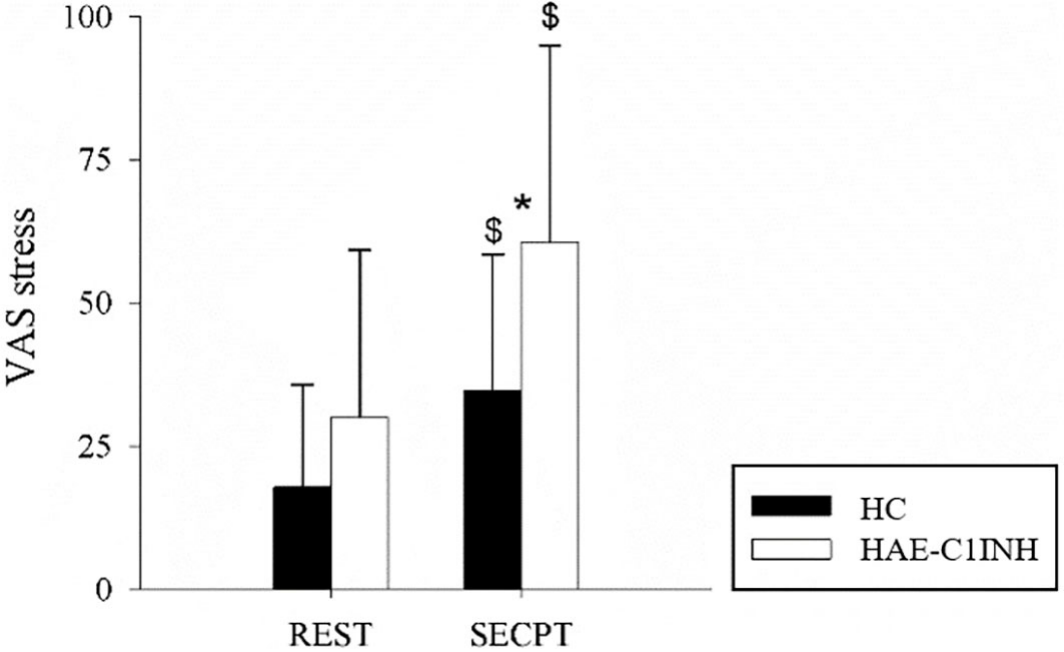
Perceived stress in HC and HAE-C1INH patients. The bar graphs show the perceived level of stress during REST and SECPT in HC (black bars) and HAE-C1INH (white bars). Data are presented as mean and standard deviation. * indicates p<0.05 vs HC, $ p<0.05 vs REST.

The results of the perceived level of stress in patients and HC are reported in **Figure 4**. The perceived level of stress during REST was equal in both patients and HC, but was higher in patients during SECPT (60.6 ± 34.3 vs 34.6 ± 23.8, p<0.05).

The perceived level of stress increased from REST to SECPT in both patients and HC. In addition, patients and HC reported similar level of unpleasantness (57.9 ± 31.4 vs 52.0 ± 30.0, respectively) and pain (59.5 ± 32.2 vs 50.7 ± 29.5) at the end of the SECPT phase as measured by the VAS scales.

## 4 Discussion

The novelty of this study lies in its multidimensional approach, integrating patients’ subjective perceptions of stress with objective cardiovascular and biochemical markers elicited by a standardized stressor, and directly comparing these responses between patients and healthy controls.

The main findings can be summarized as follows: i) HAE-C1INH patients and HC had a similar response of heart rate and arterial blood pressure to SECPT; ii) patients showed higher resting values of inflammatory biomarkers than HC; iii) inflammatory biomarkers did not vary in patients and HC after SECPT; iv) patients reported higher perceived stress in response to SECPT and were characterized by higher pain magnification compared to HC; v) none of the patients experienced attacks after the SECPT.

### 4.1 Cardiovascular control response to SECPT in HAE-C1INH patients and HC

Patients exhibited a cardiovascular response to the SECPT comparable to that of HC, suggesting that the disease does not appear to compromise their ability to cope with this specific stimulus during attack-free periods.

This finding is noteworthy, as increasing evidence in recent years has indicated the involvement of the ANS in HAE-C1INH (28, 29). Indeed, previous studies have documented that, during the early phase of an impending attack, patients display lower HR compared to the attack and post-attack phase, suggesting that an ANS impairment intervenes during the prodromal phase. The present study evaluates a different setting and demonstrates that their ability to respond to an acute stressor, such as the one induced by the SECPT remains preserved.

Specifically, patients showed increases in HR as well as in SAP and DAP during the 3-minutes cold-water hand immersion, compared to baseline values, and returned to resting levels during the 40-minutes recovery period, mirroring the responses observed in HC.

In healthy individuals, increases in SAP and DAP during the SECPT have been consistently reported in numerous studies (30–33). Conversely, the expected HR response to hand immersion remains more controversial. Not all studies have described a stable and significant HR increase across participants (30).

While some investigations reported a sustained HR elevation (33), others observed an initial marked increase followed by a gradual decline (32). Such discrepancies may be attributable to the complex and not yet fully understood mechanisms underlying ANS regulation during the SECPT. The SECPT is known to activate the sympathetic nervous system, inducing vasoconstriction (34). The individual response to the SECPT depends on both the integrity of the sympathetic noradrenergic efferent neurons and afferent baroreflex (35) pathways, and may also be modulated by individual differences in pain receptor activation (30). In the present study, the use of mean HR and AP values does not allow for definitive conclusions regarding the contribution of each underlying mechanism. Nevertheless, the key observation remains that patients and HC did not differ in their HR and AP responses to the SECPT.

### 4.2 Reaction of the inflammatory cytokines to SECPT in HAE-C1INH patients and HC

A growing number of studies reported that acute psychological laboratory stressor tests increase the concentration of the inflammatory mediators (36) and that there is an interrelationship between inflammatory biomarkers and pain (37). However, previous research on cytokine responses specifically to the SECPT remains insufficient. Our results showed that the considered inflammatory cytokines did not change across the different experimental phases either in patients or HC. The lack of detectable cytokine changes in our study can be due to several factors, such as the 40 minutes post-stress time-point being too early to observe measurable increases in plasma, with changes occurring primarily at the cellular level rather than in circulation during this period. Additionally, the cytokine response to the SECPT may have been subtle or short-lived, falling below the detection threshold or resolving before the time of measurement. Among the limited number of studies investigating cytokine responses to the SECPT, one involving healthy individuals found that those with a high subjective perception of stress showed elevated salivary IL-1β levels immediately before the SECPT (38). Since salivary cytokines may reflect local rather than systemic inflammation, we opted for plasma measurements to ensure a more reliable assessment of circulating inflammatory activity. A more recent report involving 53 potentially traumatized women and age-matched HC found significantly elevated plasma levels of IL-6, 90 minutes post-SECPT relative to baseline levels collected 15 minutes before the stressor (39) suggesting that detecting cytokine changes may require a later post-SECPT blood sampling time-point.

An important finding of our study is the increased levels of TNF- α and IL-6 in patients compared to HC, indicating an augmented inflammatory state in patients in resting condition. Notably, TNF- α and IL-1 plasma levels were found to be significantly elevated in HAE-C1INH patients during attacks and also in remission in comparison to HC in a previous study (40). In partial agreement with our data, Gramstad and colleagues (41) documented an elevated baseline thrombo-inflammatory load in HAE patients in remission, characterized by significantly higher concentrations of several pro-inflammatory cytokines, including IL1β, IL-6, TNF- α, compared to HC (41). It has to be noted that patients had a higher BMI than HC, which could also be a stimulus for chronic inflammation. Collectively, these data support the hypothesis that a chronic, low-grade inflammatory state may persist in HAE-C1INH patients even beyond the acute phase of the disease.

### 4.3 Psychological response to SECPT in HAE-C1INH patients and HC

Our results showed that patients reported significantly higher levels of anxiety symptoms than HC. This finding is consistent with previous studies conducted on similar patient populations (42). Moreover, patients exhibited higher scores on the magnification subscale of the PCS, indicating a stronger tendency to overestimate or exaggerate the perceived threat and severity of pain. Such results are expected given the chronic, debilitating, and sometimes painful nature of HAE (43), as well as the well-documented relationship between pain experience and catastrophizing in both acute and chronic conditions (44). Nonetheless, although this difference reached statistical significance, the mean PCS values in both groups remained below the clinically relevant cut-off (45), suggesting that the observed increase in pain magnification is subclinical and does not necessarily translate into heightened pain perception.

Regarding perceived stress, both patients and HC showed an increase from baseline following the SECPT, confirming that the procedure effectively induced stress in both groups. However, patients reported significantly higher perceived stress than HC immediately after the SECPT. This finding parallels the elevated pain magnification observed in the same group and aligns with prior evidence showing that magnification tendencies are positively associated with perceived stress (46). Taken together with the elevated baseline inflammatory markers observed in our study, these results further support the established link between psychological stress and inflammatory activity, which may contribute to the onset or exacerbation of angioedema attacks.

Beyond these differences in perceived stress and pain magnification, the psychological profile of patients may reflect altered stress appraisal. The SECPT combines physical discomfort with a social-evaluative threat, known to elicit stronger affective responses in individuals characterized by heightened anxiety. The higher HADS-anxiety and pain magnification scores observed in patients, although subclinical, could indicate a cognitive bias toward threat interpretation, amplifying the perception of stress even in the absence of exaggerated physiological activation. Additionally, maladaptive attentional focus on bodily sensations has been shown to reinforce sympathetic arousal and inflammatory signal through activation of the HPA axis and the SAM system (47, 48). This mechanism could partly explain the coexistence of elevated perceived stress and increased baseline IL-6 and TNF-α levels observed in these patients. Future studies should explore whether interventions targeting stress appraisal and pain catastrophizing, such as mindfulness-based or cognitive-behavioral approaches, could reduce both psychological distress and inflammatory activation, ultimately mitigating stress-related disease triggers in these patients.

### 4.4 Attacks after SECPT in HAE-C1INH patients

None of the patients experienced attacks within 72 hours following the test, although a significant difference in perceived stress immediately after the SECPT was detected when compared with HC. Several interpretations may account for this observation. First, it is possible that the procedure was not sufficiently intense or personally meaningful to induce a physiological response capable of triggering an attack. Second, LTP could have played a protective role, influencing the occurrence of attacks. Finally, it cannot be excluded that acute stress does not constitute an effective trigger for attacks in this patient population. Further studies are needed to disentangle these possibilities and to clarify the mechanisms underlying stress responsiveness in this context. Moreover, the potential contribution of chronic stress exposure to attack occurrence remains an open question deserving future investigation.

### 4.5 Limitations

Although our experimental design was complex and methodologically rigorous, the limited sample size restricts the generalizability of our findings, which should therefore be replicated in larger cohorts. Additionally, nearly all patients included were undergoing LTP treatment, whose effects on inflammatory markers and stress reactivity remain unknown. Between protocol definition and start of data collection the pharmacological scenario of LTP changed drastically: at the moment of enrolment most of the subjects who agreed to participate were receiving LTP. Unfortunately, the sample size of the present study does not allow to test the contribution of LTP. These factors should be carefully considered in future research to better isolate the impact of stress reactivity on disease expression.

Lastly, the observed difference in BMI between the groups was unforeseen and difficult to interpret unequivocally in relation to its impact on HR. Nonetheless, BMI values in both groups fall within or are close to the normal range for Western populations. Even so, future research should consider including BMI among the matching criteria for selecting control subjects, along with age and sex.

## 5 Conclusion

Our findings highlighted that the cardiovascular response to SECPT is similar between HAE-C1INH patients and HC, while patients reported significantly higher levels of perceived stress and had a greater tendency toward pain catastrophizing, particularly in the magnification dimension. Although no significant variations in cytokine levels were observed throughout the test phases, patients showed higher baseline levels of IL-6 and TNF-α compared to HC. This finding underlines a state of low-grade systemic inflammation, which may interact with psychological factors in promoting the onset of angioedema attacks.

## Data Availability

The raw data supporting the conclusions of this article will be made available by the authors, without undue reservation.
